# A household survey on screening practices of household contacts of smear positive tuberculosis patients in Vietnam

**DOI:** 10.1186/1471-2458-14-713

**Published:** 2014-07-11

**Authors:** Thuy Hoang Thi Thanh, Sy Dinh Ngoc, Nhung Nguyen Viet, Hung Nguyen Van, Peter Horby, Frank GJ Cobelens, Heiman FL Wertheim

**Affiliations:** 1 National Tuberculosis Control Programme of Vietnam, National Lung Hospital (VNTP-NLH), Hanoi, Vietnam; 2 Oxford University Clinical Research Unit (OUCRU), Hanoi, Vietnam; 3 Nuffield department of clinical medicine, Centre for Tropical Medicine UK, Oxford, UK; 4 Department of Global Health and Amsterdam Institute for Global Health and Development, Academic Medical Center, Amsterdam, Netherlands

**Keywords:** Tuberculosis, Contact screening, Case finding, Vietnam

## Abstract

**Background:**

Close contacts of tuberculosis (TB) patients are at increased risk of developing tuberculosis. Although passive contact screening guidelines are incorporated in the national TB control program, currently it is unknown how frequent close contacts are screened for TB in Vietnam. This study assesses current contact screening practices in Vietnam and determines the proportion of household contacts screened of newly registered TB patients.

**Method:**

Survey of household contacts of smear-positive TB patients (index patients) registered for treatment in 2008 in three Vietnamese cities. Households were interviewed in 2010 about screening for TB since treatment registration date of the index patient.

**Results:**

We interviewed 4,118 household contacts of 1,091 identified index cases. Contact screening mainly relied on self-referral by household contacts. Of the 4,118 household contacts, 474 (11.5%) self-referred for TB screening, while this screening proportion was only 5.5% among contacts under 5 years old (16/293). Sputum examinations were performed in 374 (78.9%) of the screened contacts. Contact screening identified 27 cases of pulmonary TB (0.7%; or 656 cases/100,000 contacts), of which 20 were detected by sputum smear.

**Conclusions:**

The low proportion of household TB contacts screened for TB illustrates the limitations of passive contact screening as currently practiced in Vietnam. Children under 5 years of age are particularly neglected with this approach. Active contact screening with fixed follow-up times of close contacts of newly diagnosed TB patients should be considered in Vietnam, particularly in case of young children and drug-resistant TB.

## Background

The World Health Organization (WHO) reported that the detected proportion of incident global tuberculosis (TB) cases is below the WHO target of 70% [1]. One of the proposed solutions is to improve the case detection rate by active and systematic screening all household contacts of pulmonary TB patients, since they are considered to be at increased risk for TB infection [2,3]. Although contact investigation is already a priority of tuberculosis control programs in many low burden and resource replete countries [4], in high burden and resource deplete areas contact screening for TB is often not performed due to the high workload and costs [5]. The recently issued *International Standard for Tuberculosis Care* states that contact investigation is an important activity and warrants more effort to ensure that persons in close contact with infectious TB patients are evaluated and managed [6].

Vietnam currently ranks 12th out of 22 countries with a high burden of TB [1]. The annual notification rate of new smear-positive pulmonary TB to the National TB Program (NTP) in recent years is about 57-58/100.000 population [7], but only accounts for 56% of new TB cases that occur annually. The remaining 44% are not diagnosed and/or diagnosed but not reported to the NTP [8]. In order to increase TB notification, the NTP utilizes both passive and active case finding strategies [9]. However in practice, due to lack of specific instructions for active case finding and insufficient resources, active case finding is rarely done. For most of the Vietnamese population, the NTP strategy relies on “passive case finding”. Passive case finding needs people to self-report with TB symptoms to primary health centers, and are then screened by sputum smear. This passive case finding strategy makes use of mass media to inform the general population about TB so that they can self-refer when symptomatic. For household contacts of infectious TB patients, a similar strategy exists as for the general population. The only difference is that TB patients and their contacts get direct information and instructions from health staff regarding when the household contacts need to come for screening. We refer to this strategy as “passive contact screening”.

This passive contact screening strategy is incorporated in the NTP guidelines and meets the minimum requirements for contact investigation as recommended by the WHO [10]. The requirements include: provide health education to TB patients and their household contacts, household visits and check for symptomatic contacts, perform sputum smear in case of prolonged cough. Health education is performed either at the health facility, where patients come to take drugs, or at home when health staff visits patients. The health education includes instructions when to seek screening, with additional instructions for children <5 years such as fever for more than two weeks or failure to thrive as their symptoms may be aspecific.

There are no data on current contact screening practices in Vietnam, while these data are essential for managing the program. Therefore, we conducted a retrospective cohort study of household contacts of patients with newly diagnosed TB in three large cities in order to assess whether they follow the NTP guideline on the following aspects: (1) screening of symptomatic household contacts, (2) screening of children <5 years, and (3) type of screening tests performed.

## Methods

### Study design and setting

We conducted structured interviews within the households of newly diagnosed TB patients who were registered for treatment with the NTP in 2008. The interviews were conducted between October and December 2010 in nine randomly selected districts of three large cities in Vietnam, three districts per each city: Hanoi, Ho Chi Minh City, and Da Nang. This study was approved by the research committee of the National Lung Hospital in Hanoi. Informed consent was obtained from TB patients and subsequently from their household contacts.

A standardized questionnaire was used to evaluate the contact screening practices of household contacts of TB patients. The interviews were conducted by local health staff of the TB program. The survey was done in the evening during working days and during daytime in weekends. In case of absence on 2 visits, the household was considered ‘lost to follow-up’. The questionnaire sought information from the patients and their household contacts about the TB screening instructions received at the time of treatment and whether the instructions were followed. Index patients and household representatives were asked about the occurrence of prolonged cough among household contacts since the diagnosis of TB in the index case and whether these contacts went for screening or received advice from health staff regarding screening. Primary caregivers provided the answers concerning children.

### Study population and definitions

The study population consisted of smear-positive TB patients registered for treatment in 2008 in one of the 9 districts mentioned above (index patients), satisfying at least one of the following criteria: (1) >1 smear- positive samples (from two different sputum specimens), or (2) one smear-positive sample and an abnormal CXR consistent with TB, or (3) a positive culture with or without positive smear. For the survey we included household contacts of notified TB cases. The following definitions were used:

•Household contact: an individual that shared the same house with the index case for a period of at least 3 months leading up to the time of diagnosis of the index case.

•Screened household contacts: a household contact who attended a public or private health facility for TB screening in the interval (days) between the day treatment was started of the index case and the interview date of the household.

•Secondary case: a household contact who was diagnosed to have TB in the interval (days) between the day start of treatment of the index case and the day of interview of the household.

•Prolonged cough: unexplained cough of more than two weeks duration occurring between the start of treatment of the index case and the interview of the household.

### Data collection and analysis

The data were collected through the NTP district coordinators. The staff of commune health stations conducted the household interviews after study specific training was provided. Data were entered into Microsoft Access software, and exported to SPSS (version 17.0) for analysis. Data quality was monitored by cross-checking 10% of the data entered in to the database against the original paper-based questionnaire. Descriptive statistics, including frequency, median, interquartile range (IQR), proportion and 95% confidence intervals (95% CIs), were performed where appropriate. Proportions were compared using the chi-squared test. P–values below 0.05 were considered significant (two-sided).

## Results

### Demographic and clinical characteristics of study population

We interviewed the household contacts of 1,091/1,215 (89.8%) smear-positive TB patients registered in 2008 in the nine selected districts. Households of 124 index TB patients (10.2%) did not participate in the study because of the death of the index case, absence at the time of interview, or having moved to another address.

**Figure 1:**
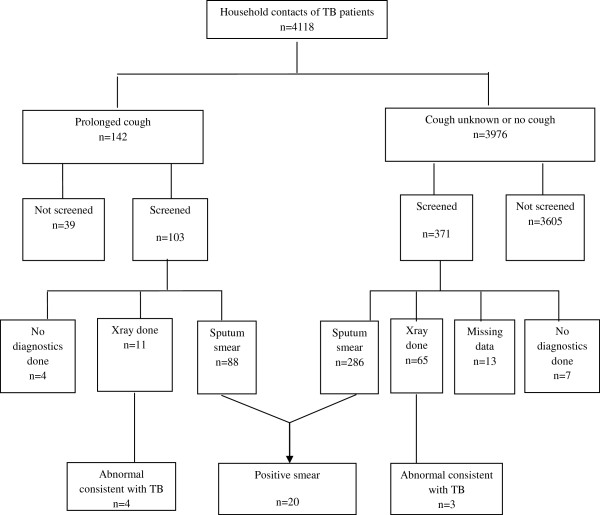
Flow chart of tests taken for household contacts of smear-positive TB patients in three cities of Vietnam, 2008–2010.

Demographic and clinical characteristics of index TB patients and their household contacts are described in Table 1. The male-to-female ratio was approximately 3:1. Of the 109 index patients, 90 (8.2%) were HIV positive and 77 (7.1%) had a history of drug abuse. Of 4118 household contacts, 293 (7.1%) were children under 5 years of age at the time of the interview, and 142 had prolonged productive cough at any time between registration of index patient and the interview (3.4%; 95% CI 2.9-4.0%; Figure 1). Of 1,091 index patients, 1,017 (93.2%) stated that none of their family members, other than the index case, had ever had a diagnosis of TB.

**Table 1:** Characteristics of study population

**Category**	**Characteristics**		**n**	**%**
Index TB patients				
	Sex	Male	816	74.8
		Female	275	25.2
	Age	<5	0	0.0
		5–14	0	0.0
		>15	1091	100.0
Household contacts				
	Age	<5	293	7.1
		5–14	631	15.3
		>15	3194	77.6
Household contacts who went for TB screening				
	Sex	Male	170	35.9
		Female	304	64.1
	Age	<5	16	3.4
		5–14	31	6.5
		>15	427	90.1
Household contacts with smear examination				
	Sex	Male	132	35.3
		Female	242	64.7
	Age	<5	2	0.5
		5–14	13	3.5
		>15	359	96.0
Secondary TB cases detected from contacts (any test)				
	Sex	Male	13	48.1
		Female	14	51.9
	Age	<5	1	3.7
		5–14	2	7.4
		>15	24	88.9
Secondary TB cases detected from contacts (positive smear)				
	Sex	Male	11	55.0
		Female	9	45.0
	Age	<5	0	0.0
		5–14	2	10.0
		>15	18	90.0

TB = tuberculosis.

### Household screening

**Figure 2:**
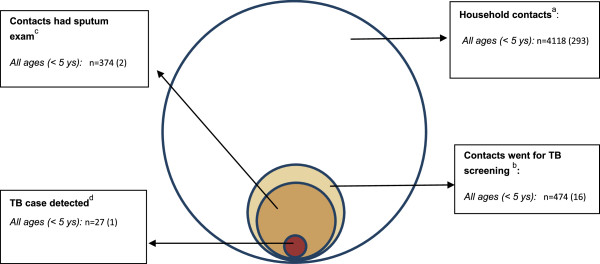
Venn diagram for screening of household contacts of smear-positive TB patient in three cities of Vietnam, 2008–2010.

The median number of household contacts per index patient was 3.8 (IQR: 3–5). During the interval between the registration of the index case and the interview, at least one household contact was screened for 335/1,091 (30.7%; 95% CI: 28.0-33.4%) index patients. A total of 474/4,118 (11.5%; 95% CI: 10.5-12.5%) of all household contacts sought TB screening, including 16/293 (5.5%; 95% CI: 2.9-8.1%) contacts aged under 5 years (Figure 2). The rate of screening was higher (p-value <0.05) among those with prolonged cough (103/142; 72.5%; 95% CI: 65.2-79.9%) than among those without prolonged cough and/or cough unknown (371/3976; 9.3%; 95% CI: 8.4-10.2% Figure 1). Detailed information regarding the examinations performed was available for 461/474 (97%) individuals who went for TB screening. Smear examination was done for 374/461 contacts (81.1%), 76 (16.5%) contacts only had CXR and 11 (2.4%) had neither sputum nor CXR examination (Figure 1). Of 103 contacts with prolonged productive cough who were screened, 88 were tested by direct sputum smear examination (85.4%; 95% CI: 78.6-92.3%), 11 (10.7%; 95% CI: 4.7-16.6%) only by CXR and 4 (3.9%; 95% CI: 0.2-7.6%) had no specific TB testing. There was no significant difference in the proportion of contacts that went for TB screening by smear grade of the index case (p value = 0.23, Table 2).

**Table 2:** Screening for contacts grouped by sputum grade of index patients

**Positivity grade of index cases**^**a**^	**No of Index patient**	**No of contacts**	**Contacts per index case**	**Contacts went for screening n (%)**		**Secondary cases with positive smear; n (%)**	
3+	144	563	3.9	75	13.3	3	4.0
2+	183	699	3.8	75	10.7	3	4.0
1+	610	2249	3.7	265	11.8	9	3.4
Scanty	154	607	3.9	59	9.7	5	8.5
Total	1091	4118	3.8	474	11.5	20	4.2

^a^Based on the International Union Against Tuberculosis and Lung Disease (IUATLD)-recommended grading of sputum smear microscopy results.

Just 103 (21.7%) symptomatic contacts that went for screening had prolonged productive cough, and the remaining 371 (78.3%) had no prolonged cough and/or cough unknown. 39 contacts with prolonged cough did not go for screening. Reported reasons for not going for screening included: busy earning a living, feeling better after self-medication with over-the-counter drugs, and afraid of unaffordable treatment cost.

Among the 4,118 household contacts, 27 secondary TB cases were detected (detection rate: 0.7% or 656 patients/100,000 contacts), of which 13 were male. One TB case was detected among children under 5 years of age (Table 3). 20 contacts with TB were diagnosed by positive sputum smear and 7 by CXR (Figure 1).

**Table 3:** TB case detected among contacts of smear positive TB patients

**Contact by characteristic**		Contacts went for screening*	**Contacts with sputum examination**	Total TB cases detected**	**Smear positive TB cases detected**
***Types***	***Number***				
All household contact	4118	474	374	27	20
Contacts with prolonged cough	142	103	88	21	17
Contacts without prolonged cough or cough unknown	3976	371	286	6	3
Children under 5 years of age	293	16	2	1	0

*Includes contacts who came to public or private health facility for screening.

**Either with smear-positive or smear-negative tuberculosis detected from contacts.

TB = tuberculosis.

The index patients had a median duration of 2 days (IQR: 1–4 days) from diagnosis to treatment. The median duration from treatment registration of the index case to screening of contacts was 29 days (IQR: 9–65 days), and for TB positive contacts this was 97 days (IQR: 37–316 days).

Most index patients (n = 722; 66.2%) were aware that any adult contact with prolonged cough should go for TB screening. Sixty-nine (6.3%) index patients thought screening was not required for household contacts and only 68 (6.2%) were aware of the risks for children under 5 years of age in their household.

## Discussion

This study shows that with the current system of passive contact screening for TB, ~10% of all household contacts are screened, and just 5.5% of children under 5 years of age. These rates are low and need to be improved as studies show that up to 22% of household contacts in high prevalence countries have TB [5]. The low proportion of household contacts screened is likely a consequence of the passive nature of the strategy, which mainly relies on typical clinical symptoms being experienced by the contact and persons seeking appropriate health care on their own initiative [11]. In this study, several contacts did not go for TB screening even when they experienced prolonged cough. Reported reasons for this are related to unawareness about the need for screening and fear of costly diagnosis and treatment. Although the vast majority of TB patients (66%) knew about the need for investigating contacts, one-third were not aware that contacts with prolonged cough need to be screened. Furthermore, very few of the TB patients (6.2%) knew that young children need to be screened in case of suspect symptoms, and as a result children under 5 years of age are usually ignored. There is a need to evaluate the effectiveness of health education materials and methods being used in the NTP to optimally utilize them so that contact tracing can be performed more effectively.

Currently the NTP only focuses on contacts that self-report to a health facility with a persistent productive cough for at least 2 weeks [9,12]. A large TB prevalence survey in 2006–2007 in Vietnam revealed that 47% of TB cases had other symptoms than cough or only chest X ray abnormalities [8]. Thus half of the patients may be missed if the program only focuses on cough. Another issue that may contribute to under-diagnosis is the method of screening for TB. According to the Vietnam NTP guidelines, direct smear examination is the main diagnostic test provided for people who present themselves at a health facility with symptoms suggestive of TB. However, in this study, 15% of TB contacts with a prolonged productive cough who went for screening were not examined by sputum smear.

In order to improve TB case detection, systematic contact investigations need to be considered for implementation in low income and middle income countries with endemic TB levels [13]. The potential yield of TB cases from contact investigation in high- and low-incidence settings has been reviewed previously [4,5]. In a meta-analysis, the prevalence of TB among all household contacts is estimated to be 3.1% [4]. In high-prevalence countries, up to 22% of household contacts have active tuberculosis [5]. Furthermore, in settings with high proportion of HIV-positive TB cases, active screening among household contacts yields nine times more TB cases as compared to passive case finding [14]. Recently, programs are starting to utilize resources for targeted screening of contacts of MDR-TB patients, HIV positive contacts and children [15].

This study has several limitations. We collected data over a limited time period (from index patients registered for treatment in 2008 up to the time of the interview in October to December 2010. Also the retrospective nature of this study may result in recall bias. We may have therefore missed TB disease among contacts that either developed disease after our study or may nor recall correctly. As the questions were simple and straightforward on a major health issue in the household, we believe we were able to minimize recall bias. Furthermore, several studies have shown that the highest proportion of TB cases among contacts are detected in the first two years after exposure and therefore we think only few may have developed disease after our study [4,16].

Furthermore, it is difficult to conclude whether the detected TB cases among contacts went for screening due to the health education provided to them at the time of the index case diagnosis or due to other reasons. However, as most TB cases among contacts were diagnosed relatively shortly after the diagnosis and treatment of the index case (~3 months) we believe that the majority went for screening as a result of instructions provided to them at the health education. Another limitation is that no rural areas were included in this study and our findings only apply to urban areas. There may be better screening practices in urban areas due to higher education levels and knowledge about TB, better access to health facilities and more resources for TB diagnostics.

Another limitation was that the assessment of NTP guidelines regarding contact screening was done by health staff, who are also part of implementing these NTP guidelines (i.e. TB unit staff). This may have biased the results. However, the interviewers were trained in survey techniques and were instructed not to be concerned about ‘right’ or ‘wrong’ answers. Furthermore, the study progress was supervised by two independent supervisors (FC and HW). Since the results are not supportive of the current program, we believe we have been successful in minimizing bias.

## Conclusions

This study shows that the proportion of household contacts of smear-positive tuberculosis patients screened for TB under the current passive screening approach of the Vietnam National TB program is very low compared with prevalence of TB among contacts in high burden countries. Better health information and instructions need to be provided to contacts of TB patients to improve their health seeking behavior in case of possible TB symptoms, with a focus on vulnerable groups like HIV-positive contacts, contacts of drug resistant TB cases and contacts under 5 years of age. Special attention is needed to provide guidance when to seek TB screening for children. Contact investigation should be conducted more actively and systematically starting by recording the contacts information for management and follow-up. Active contact tracing of close contacts of newly diagnosed TB patients should be considered in Vietnam, particularly in case of young children and drug-resistant TB. Studies that assess how this can be efficiently done are required.

## Competing interest

The authors declare that they have no competing interests.

## Authors’ contributions

HW, PH, HTTT conceptualized and designed the study. HTTT was responsible for data collection, entry, analysis and interpretation of data. HTTT wrote the first draft of the paper with critical advices provided by HW and FC. All authors contributed to review , read and approved the manuscript as submitted.

## Pre-publication history

The pre-publication history for this paper can be accessed here:

http://www.biomedcentral.com/1471-2458/14/713/prepub
